# Application of the Shellac Cloth or Poroplastic Spinal Jacket

**Published:** 1879-02

**Authors:** 


APPLICATION OF THE SHELLAC CLOTH OR POROPLASTIC SPINAL JACKET.—Mr. J. C. Hutchinson, of the Brooklyn Orthopædic Infirmary recommends the following material and mode of application in cases of spinal caries, and of diseases of the hips and knee-joints. The felt, or, as it is called, shellac cloth, should be of heavier kind than that employed by hatters. Then the patient, being suspended in a Sayre's swing, the following measurements should be taken:—1. From a point one and a half inches to one side of the linea alba, on a line with the anterior superior spinous process of the ilium, around the back of the pelvis, to a corresponding point on the opposite side. 2. From a point one and a half inches to one side of the middle of the sternum around the thorax, to a corresponding point on the opposite side. 3. From the seventh cervical vertebræ to the top of the sacrum: slits are cut in the lower edge of the splint an inch long and two or three inches apart, so as to allow the lower part to mould itself more accurately to the pelvis, and thus get a better base of support. It should also be cut away under the arms. The chest is now covered by a well-fitting woolen shirt or flannel bandage, the patient being suspended. The felt, made pliable by dry heat or steaming, or by immersion in very hot water, is quickly applied, and covered with a bandage from below upward very firmly, so as to mould it to all the inequalities of the surface. The splint almost immediately regains its inflexibility. It may then be removed, trimmed up, and holes punched along the front edge and reapplied. Two brace-strips may be attached to the top edge of the splint, which cross over the shoulders and buckle in front. The splint may be made more comfortable in hot weather by perforating here and there with a punch. The splint for a child weighs only eight to twelve ounces—very much lighter than a plaster of Paris jacket—is comfortable, cleanly and more easily applied, while it fulfils all indications.—*Lancet, Aug.* 31, 1878.

